# Specific Magnetic Isolation of *E6 HPV16* Modified Magnetizable Particles Coupled with PCR and Electrochemical Detection

**DOI:** 10.3390/ijms17050585

**Published:** 2016-05-05

**Authors:** Ana Maria Jimenez Jimenez, Branislav Ruttkay-Nedecky, Simona Dostalova, Ludmila Krejcova, Petr Michalek, Lukas Richtera, Vojtech Adam

**Affiliations:** 1 Department of Chemistry and Biochemistry, Mendel University in Brno, Zemedelska 1, CZ-613 00 Brno, Czech Republic; anuskajj@hotmail.com (A.M.J.J.); simona1dostalova@gmail.com (S.D.); lidakrejcova@seznam.cz (L.K.); petrmichalek85@gmail.com (P.M.); oliver@centrum.cz (L.R.); 2 Central European Institute of Technology, Brno University of Technology, Purkynova 123, CZ-612 00 Brno, Czech Republic; brano.ruttkay@seznam.cz

**Keywords:** electrochemistry, human papillomavirus, magnetic isolation, nucleic acid detection, magnetizable particles, PCR

## Abstract

The majority of carcinomas that were developed due to the infection with human papillomavirus (HPV) are caused by high-risk HPV types, HPV16 and HPV18. These HPV types contain the E6 and E7 oncogenes, so the fast detection of these oncogenes is an important point to avoid the development of cancer. Many different HPV tests are available to detect the presence of HPV in biological samples. The aim of this study was to design a fast and low cost method for HPV identification employing magnetic isolation, polymerase chain reaction (PCR) and electrochemical detection. These assays were developed to detect the interactions between *E6-HPV16* oncogene and magnetizable particles (MPs) using commercial Dynabeads M-280 Streptavidin particles and laboratory-synthesized “homemade” particles called MANs (MAN-37, MAN-127 and MAN-164). The yields of PCR amplification of *E6-HPV16* oncogene bound on the particles and after the elution from the particles were compared. A highest yield of E6-HPV16 DNA isolation was obtained with both MPs particles commercial M-280 Streptavidin and MAN-37 due to reducing of the interferents compared with the standard PCR method. A biosensor employing the isolation of *E6-HPV16* oncogene with MPs particles followed by its electrochemical detection can be a very effective technique for HPV identification, providing simple, sensitive and cost-effective analysis.

## 1. Introduction

The human papillomavirus 16 (HPV16) is considered a “high-risk” human papillomavirus, based on its high potential for oncogenesis [1,2]. This type of virus encodes three domains, a non-coding regulatory region; a late region, which encodes the capsid proteins (L1 and L2); and an early region, which encodes the viral DNA replication proteins (E1, E2, E4 and E5) and the oncoproteins E6 and E7, which promote cellular proliferation or inhibit cell death, thus contributing to the development of carcinogenesis [3,4,5].

The E6 and E7 oncogenic proteins are necessary for the malignant conversion and they have different strategies against the immune system to promote cellular transformation and the carcinogenesis in squamous cells of human skin. They also produce transformation of established cell lines, immortalization of primary cell line, transmembrane signaling and regulation of chromosomal stability [6,7].

Regulation and function of these two oncoproteins, can explain the molecular mechanism of HPV related carcinogenesis. E6 and E7 proteins that inactivate the p53 and pRb pathways can result in the increase of cell proliferation, accumulation of mutations and thus causing carcinoma development [3,8].

**Figure 1:**
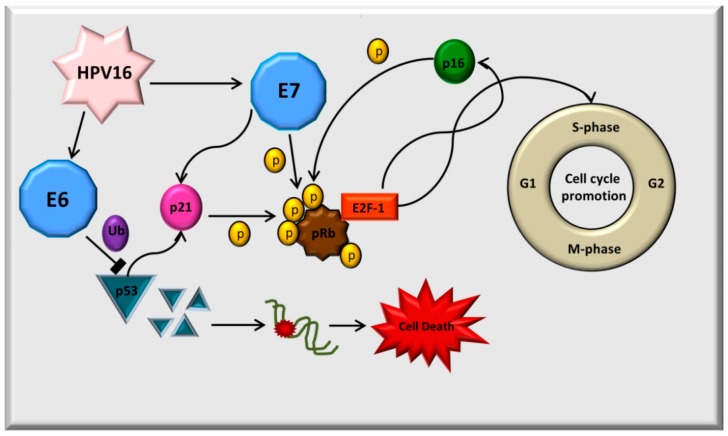
Main molecular mechanisms of oncogenesis induced for E6 and E7 proteins of human papillomavirus 16 (HPV16).

The E6 oncoprotein consists of 158 amino acid residues and contains two zinc-binding domains, similar to the E7 oncoprotein. E6 oncoprotein can join with the p53 tumor suppressor protein and promote its degradation by the cellular ubiquitination, perturbing the control of cell cycle progression and leading to the increased tumor growth. The major role of E6 is the degradation of p53, reducing the cell’s ability to respond to DNA damage (Figure 1). The E6 oncoprotein can also have oncogenic activities independent of p53. Further, the E6 oncoprotein can activate the telomerase enzyme, which is highly functional in human cancers and immortalized cell lines [9].

The E7 oncoprotein has 100 amino acid residues. The E7 oncoprotein binds to the under-phosphorylated pRb and inactivates it. The biological function of pRb is to bind to the the E2F transcription factor 1 (E2F-1), associated with DNA synthesis. The E7 oncoprotein disrupts this interaction by phosphorylation of Rb; the E2F-1 is released and can induce the transcription of the S-phase genes. Inactivation of Rb and inhibition of the feedback mechanism leads to the overexpression of the p16 protein, which stimulates the replication and cell-division in keratinocytes during carcinogenesis. The E7 oncoprotein also induces abnormal centrosome duplication, abnormal mitoses, aneuploidy and genomic instability [10] (Figure 1).

The commonly used methods for analysis of nucleic acids include electrophoresis [11,12], ultraviolet-visible spectrometry [13,14], mass spectrometry [15], immunology [16,17] and circular dichroism spectroscopy [18,19]. Besides these methods, the electrochemical techniques can also be employed [20]. The electrochemical techniques are promising for rapid and sensitive determination of nucleic acids [20]. The principle of the electrochemical detection of nucleic acids is based on the detection of redox signals, mediated by electro-activity of bases (adenine, guanine and cytosine). Square wave voltammetry (SWV), cyclic voltammetry (CV), elimination voltammetry (EV) and differential pulse voltammetry (DPV) can be employed in the detection of nucleic acids [21,22,23]. SWV in combination with the adsorptive transfer stripping (AdTS) technique increased sensitivity and decrease sample consumption [24]. In this technique, the working electrode is dipped into a microliter-sized drop for direct adsorption of the nucleic acid. Using these techniques a successful detection of nanogram quantities of nucleic acid was achieved [21]. In this study, AdTS SWV was selected as one of the cornerstone methods for nucleic acid detection.

On the other hand, in order to detect HPV infection, many different tests have been used. One of the most common is the polymerase chain reaction (PCR) technique. PCR represents a highly-sensitive and cost-effective method for HPV detection. In theory, it can be used to detect as little as one copy of a DNA sequence and can be utilized in paraffin-embedded tissue or fresh tissue from biopsies [10,25]. However, the PCR techniques have a number of drawbacks: they have lower specificity, they do not allow distinction between HPV that is present in the neoplastic and non-neoplastic cells, they cannot distinguish between episomal and integrated HPV DNA and they also cannot measure the viral load [26,27].

In this work, a novel approach for HPV detection is proposed based on the amplification of *E6-HPV16* gene using magnetizable particles (MPs) modified by primers coupled with electrochemical and electrophoretic gel detection of the isolated nucleic acid in combination with PCR method. The main aim of this study was to design and optimize a method for the PCR amplification of target DNA, followed by its verification by electrochemistry and gel electrophoresis. Beside this, another task was the optimization of the electrochemical detection of cytosine-adenine (CA) peak in amplified target DNA (*E6-HPV16* gene). Our assay was developed in two parts: (i) isolation of *E6-HPV16* oncogene using MPs; and (ii) electrochemical detection of isolated E6-HPV16 DNA. The presented technique provides simplicity, high sensitivity, speed, and cost effectiveness and could therefore be utilized in an electrochemical biosensor, for detection of viruses [28,29].

## 2. Results

### 2.1. Polymerase Chain Reaction (PCR) Optimization of E6-HPV16 (Human Papillomavirus 16) Oncogene

**Figure 2:**
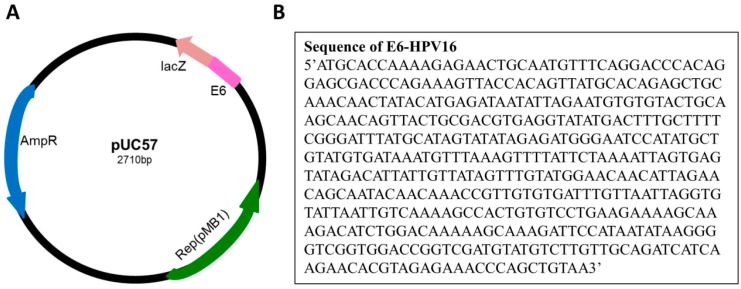
(**A**) Scheme of the pUC-57 vector with cloned *E6* gene of HPV16. The construction of human E6-HPV16-pUC57 plasmid was purchased from Genewiz Company (GENEWIZ, South Plainfield, NJ, USA); (**B**) Nucleotide sequence of *E6-HPV16* gene.

**Figure 3:**
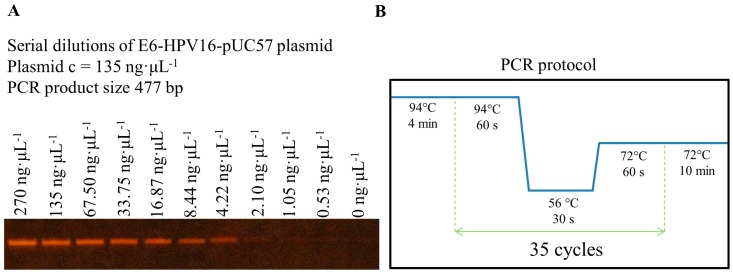
Optimization of template E6-HPV16-pUC57 plasmid concentration and scheme of standard PCR conditions: (**A**) finding the optimal concentration of DNA template for polymerase chain reaction (PCR); and (**B**) PCR protocol.

Isolation of the *E6-HPV16* gene was obtained after PCR amplification of E6-HPV16-pUC57 plasmid. Fifty nanograms of the synthetic plasmid were added to *Escherichia coli* (*E. coli*) TOP 10 chemical competent cells. After the chemical transformation, the full-length clones of *E6-HPV16* gene were obtained by PCR amplification of DNA template, using a set of primers flanking the complete open reading frame. The primers were designed by online Primer 3 (v. 0.4.0) software (http://bioinfo.ebc.ee/mprimer3/) (Figure 2). The positive transformants were confirmed by PCR screening (data not shown). The E6-HPV16-pUC57 plasmid was purified using the Qiagen Miniprep Kit (Qiagen, Germantown, MD, USA). The concentrations of E6-HPV16-pUC57 plasmid template and other PCR chemicals were optimized for obtaining higher yield of *E6-HPV16* gene amplification. Starting with the DNA plasmid concentration of 135 ng·µL^−1^, serial dilutions were prepared and the influence of increased and decreased concentrations of PCR chemicals on the amplification yield were evaluated. Optimization of the concentration of the individual components of master mix were prepared according to the following protocol: standard conditions (200 µM dNTPs, 1.5 mM MgCl_2_, 0.4 µM primers, and 0.5 units of Taq polymerase per 25 µL); increased conditions (225 µM of dNTPs, 1.6 mM MgCl_2_, 0.5 µM of each primer and 0.55 units of Taq polymerase per 25 µL); and decreased conditions (175 µM of dNTPs, 1.4 mM MgCl_2_, 0.3 µM of each primer and 0.45 units of Taq polymerase per 25 µL). The PCR amplification was performed for 35 cycles with annealing temperature of 56 °C (Figure 3).

Optimization of PCR amplification and electrophoretic detection of *E6-HPV16* oncogene cloned in pUC57 plasmid was evaluated prior to starting the rest of the experiments to evaluate the optimal conditions for PCR. The highest observed yield was achieved using 270 ng of E6-HPV16-pUC57 plasmid template and using the lowest concentrations of the PCR chemicals (175 µM of dNTPs, 1.4 mM MgCl_2_, 0.3 µM of each primer and 0.45 units of Taq polymerase per 25 µL).

### 2.2. Isolation of E6-HPV16 Gene Using Commercial MPs with PCR Detection

**Figure 4:**
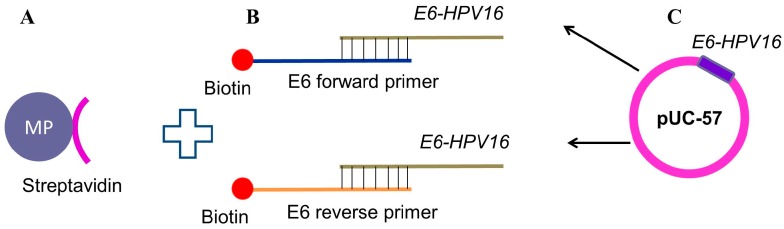
Scheme of DNA nanoconstruct of biotin-modified oligonucleotides bound to *E6-HPV16* oncogene joined to streptavidin modified MPs : (**A**) 100 µL (10 mg·mL^−1^) of commercial Dynabeads M-280 Streptavidin MPs; (**B**) E6-HPV16 complementary oligonucleotides biotinylated (forward and reverse) (20 µL, 100 µM) using Biotin 3′ end DNA Labeling Kit (Thermo Scientific, Waltham, MA, USA) were successfully conjugated with Dynabeads; and (**C**) E6-HPV16 DNA was amplified from E6-HPV16-pUC57 synthetic plasmid by PCR, which was subsequently purified and conjugated with the nanoconstruct.

In our study, streptavidin-modified commercial MPS (M-280 Streptavidin Dynabeads (Invitrogen, Waltham, MA, USA)) were used for the isolation of *E6-HPV16* gene, after conjugation with oligonucleotides labeled with biotin. The forward and reverse oligonucleotides were complementary to the *E6-HPV16* gene and they were biotinylated in the 3′ end. After calculating the binding capacity of M-280 Streptavidin Dynabeads commercial MPs the biotinylated oligonucleotides conjugated with the particles, followed by *E6-HPV16* gene were previously amplified by PCR from E6-HPV16-pUC57 plasmid and purified from the PCR mixture. Figure 4 shows the scheme of *E6-HPV16* gene isolation using this approach.

**Figure 5:**
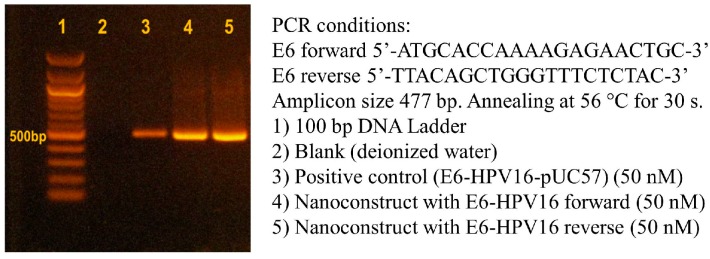
Comparison of PCR assay between E6-HPV16 DNA isolated from synthetic plasmid and E6-HPV16 DNA isolated using commercial M-280 MPs, which they were used to make a PCR with set of E6 primers. The DNA nanoconstructs with commercial MPs particles showed 2.6-fold higher fluorescence of Ethidium bromide (EtBr) stained E6-HPV16 bands than the positive control using synthetic plasmid E6-HPV16-pUC57. Conditions for gel electrophoresis were as follows: 1% agarose gel, 1% TAE buffer.

The measurements were performed after the separation of the DNA nanoconstruct from commercial M-280 MPs by external magnetic field. The presence of DNA nanoconstruct was confirmed by spectrophotometry and it was immediately utilized for PCR with electrophoretic detection, which subsequently revealed successful isolation of *E6-HPV16* gene fragment of 477 bp. A 2.6-fold higher fluorescence of EtBr-stained E6-HPV16 bands was observed in case of isolation with commercial M-280 MPs when compared to direct PCR amplification of E6-HPV16-pUC57 plasmid (same concentration of DNA for all samples were used in the PCR) (Figure 5).

### 2.3. Isolation of E6-HPV 16 Gene in a Plasmid Using Different “ Homemade” MPs with PCR Amplification and Detection

**Figure 6:**
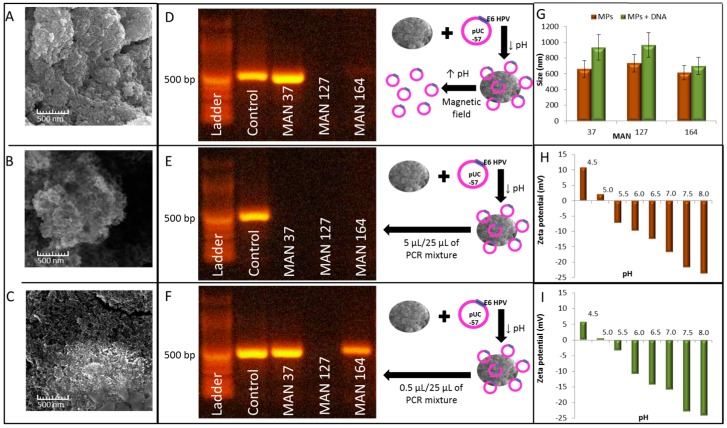
Isolation and detection of E6-HPV16-pUC57 plasmid using different “homemade” magnetiyable particles (MPs): (**A**–**C**) SEM microphotographs of MPs used in DNA isolation procedure (MAN-37, MAN-127 and MAN-164 respectively); (**D**) PCR amplification of *E6-HPV16* gene in pUC-57 plasmid after elution from MPs; (**E**) PCR amplification of *E6-HPV16* gene from 5 µL of MPs with bound DNA; (**F**) PCR amplification of *E6-HPV16* gene from 0.5 µL of MPs with bound DNA; (**G**) the average size of MAN-37, 127 and 164 before and after binding with DNA; (**H**) the dependence of MAN-37 zeta potential on pH; and (**I**) the dependence of zeta potential of MAN-37 with bound DNA on pH.

An isolation of E6-HPV16-pUC57 plasmid was performed using three different MPs prepared in our laboratory: MAN-37 with 3-aminopropyltriethoxysilane (APTES) coating, MAN-127 with PVP coating and MAN-164 with calcium nitrate and sodium triphosphate coating. Figure 6A–C shows SEM (Scanning Electron Microscopy) microphotographs of used particles. These particles were used to isolate 100 ng of E6-HPV16-pUC57 plasmid and the eluate from particles was used for subsequent PCR amplification with EtBr detection after gel separation (Figure 6D). The gel shows successful isolation with subsequent detection of plasmid only in case of used MAN-37 and a very slight band in case of used MAN-164. After this, a PCR of plasmid still attached on particles was performed, using 3 times lower amount of plasmid as was determined in the eluate (Figure 6E). However, the PCR of plasmid left over on particles did not work. In the next step, we used 30 times lower amount of plasmid in comparison with the amount in eluate (Figure 6F). Distinct bands can be observed both in the case of isolation with MAN-37 and MAN-164. The intensity of band was comparable to the one from eluate in case of MAN-37.

The average zeta potential is more negative at all pH values in the case of particles after binding of DNA, corresponding to the negative charge of DNA molecules.

### 2.4. Optimization of Electrochemical Detection of of Cytosine-Adenine (CA) Peak of the Product of Amplification

**Figure 7:**
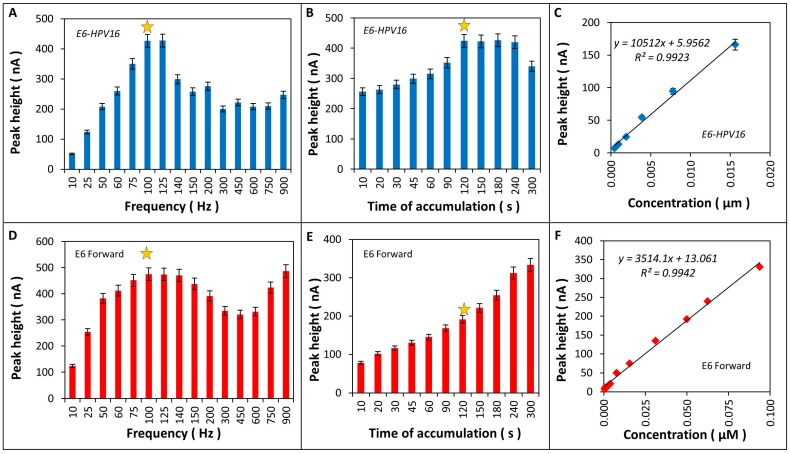
Optimization of electrochemical detection of *E6-HPV16* gene and E6 forward primer (E6 Fw) were as follows: (**A**) frequency; (**B**) time of accumulation; and (**C**) calibration curve for *E6-HPV16* gene; (**D**) frequency; (**E**) time of accumulation; and (**F**) calibration curve for E6 forward primer. All electrochemical measurements were provided by AdTS SWV in acetate buffer, pH 5.0, with an initial potential 0.0 V, end potential −1.7 V, amplitude 0.025 V, step potential 0.005 V. 100 Hz of frequency and 120 s of time of accumulation were selected as optimal conditions (yellow asterisks).

Electrochemical detection of *E6-HPV16* gene and E6 forward primer (E6fw) by AdTS SWV was performed. *E6-HPV16* gene was amplified and purified from pUC57 plasmid, where it was cloned previously (GENEWIZ, South Plainfield, NJ, USA) and E6 forward primer was synthetized by Invitrogen. Two of the detection parameters (frequency and time of accumulation) were optimized. The influence of frequency on the CA peak height was tested in the range of 10 to 900 Hz, as the optimum 100 Hz was selected separately for *E6-HPV16* gene (Figure 7A) and E6 forward primer (Figure 7D) because they were differentiated from each other in length and structure (*E6-HPV16* gene was double stranded DNA of 477 bp and E6 forward primer was single stranded DNA of 20 bp). Other optimized parameter was the time of accumulation and its influence on CA peak height was observed in the range 10 to 300 s, as the optimum 120 s was selected for both *E6-HPV16* gene (Figure 7B), as well as E6 forward primer (Figure 7E). Under the optimized conditions, calibration curves for both sequences (*E6-HPV16* gene and E6 forward) were provided (Figure 7C, F).

### 2.5. Detection of CA Peak in the Product of PCR Amplification of E6-HPV16 Gene

**Figure 8:**
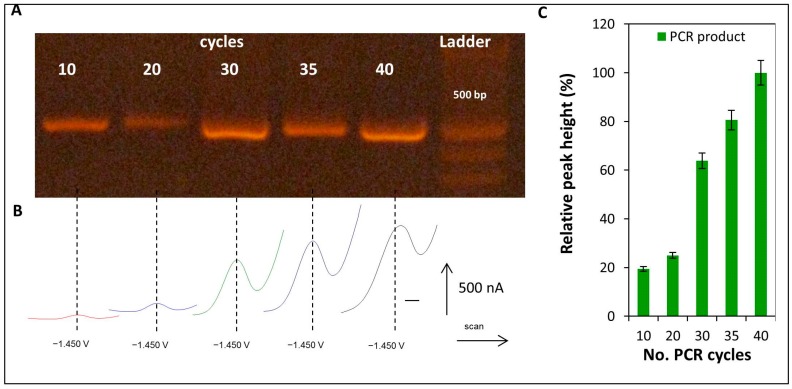
(**A**) Gel electrophoresis and (**B**) electrochemical analysis of PCR product (PCR of E6-HPV16-pUC57 plasmid set of E6 primers) obtained after 10, 20, 30, 35, and 40 PCR cycles. Expected size was 477 bp, corresponded to *E6-HPV16* gene. Gel electrophoresis conditions: 1% agarose gel, 1% TAE buffer. Electrochemical conditions: See optimized conditions from Figure 7B; (**C**) Dependence of the relative CA peak height on the number of PCR cycles.

The electrochemical analysis of *E6-HPV16* gene products after different number of PCR cycles was provided. Five PCR-amplified samples were used, obtained using 270 ng of the E6-HPV16-pUC57 plasmid as a template in each sample. The PCR conditions were previously optimized (see Chapter 2.1 Polymerase Chain Reaction (PCR) Optimization of *E6-HPV16* (Human Papillomavirus 16) Oncogene). PCR products obtained after 10, 20, 30, 35, and 40 cycles of amplification of E6-HPV16-pUC57 plasmid used as a template were studied by electrochemistry. AdTS SWV under optimized conditions (for details see Section 4.9.1 Optimization of Electrochemical Detection of CA peak in Product of Amplification) was employed. Real CA peak voltammograms (peak position −1.45 ± 0.05 V) are shown in Figure 8B and the obtained results were compared with results from gel electrophoresis (Figure 8A). Dependence of the relative CA peak height on number of PCR cycles is shown in Figure 8C.

An exponential correlation between the number of the PCR cycles and the relative peak heights was showed, thereby confirming that a minimum of 30 PCR cycles was needed to obtain relevant detection of the HPV samples.

### 2.6. Electrochemical Characterization of E6-HPV16 Gene as a Product after Magnetic Isolation

Two different nanoconstructs using M-280 streptavidin-coatedmagnetizable particles modified by biotinylated oligonucleotide (forward and reverse primers) complementary to *E6-HPV16* oncogene were prepared. The electrochemical measurements of *E6-HPV16* gene were performed after the obtaining of the eluted DNA from the nanoconstruct using high temperature and separation from commercial MPsparticles by external magnetic field. Two *E6-HPV16* gene products, one amplified by forward oligonucleotide and the other amplified by reverse oligonucleotide, were obtained.

**Figure 9:**
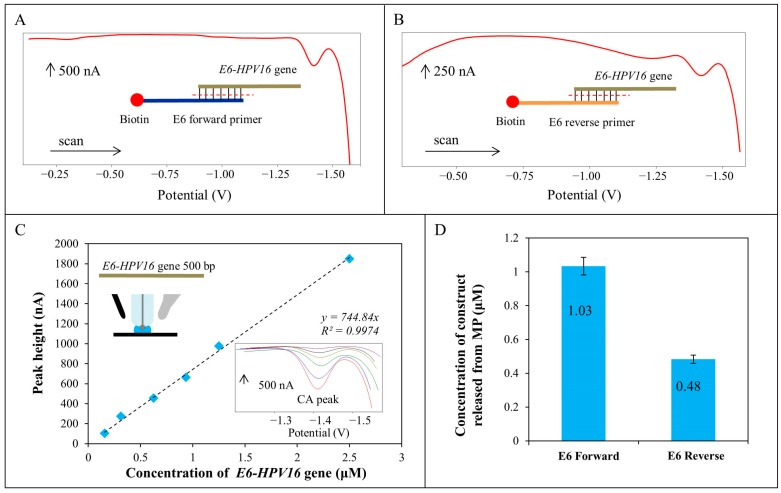
(**A**) Typical SWV voltammograms of E6 forward primer construct released from commercial M-280 MPs; (**B**) compared with E6 reverse primer construct released from commercial M-280 MPs; (**C**) calibration curve of *E6-HPV16* gene in concentration range from 0.15 to 2.5 µM, real CA peak voltammograms and scheme of accumulation of sample on surface of working electrode are inserted; and (**D**) concentration of *E6-HPV16* gene construct isolated by commercial M-280 MPs by two different primers (E6 forward and E6 reverse). The up arrows show the scale for each voltammograms.

For the characterization of products of the *E6-HPV16* gene magnetic amplification AdTS SWV method under optimized conditions was employed. Impact of forward and reverse primers on the peak height and voltammograms shape is shown in Figure 9A,B. Concentration of the E6-HPV16 construct released from commercial M-280 MPs was two times higher in the case of using of forward primer, due to the more effective hybridization between oligonucleotide and magnetic particles that was depended on many factors, including nucleotide sequence, temperature, salt concentration and the time of hybridization.

## 3. Discussion

Nowadays, nanotechnologies have many advantages and many outstanding possibilities for their application in the detection of diseases. Biosensing instruments based on magnetic beads separation are becoming the leader in technological research and innovation because of the fact that they can recognize a very low concentration of target molecules, and also because of their high specificity and easy handling. Currently, this method has a great potential for the detection of pathogens, viral agents, nucleic acids, in combination with other detection methods like microfluidic systems, PCR, immunoassays, electrochemical techniques, *etc.* [28,30].

In this study, the electrochemical detection of *E6-HPV16* gene cloned in pUC57 plasmid was optimized. The *E6-HPV16* gene was isolated using magnetizable Dynabeads M-280 Streptavidin (commercial) particles and magnetizable “homemade” MAN-37, MAN-127 and MAN-164 particles (prepared in the laboratory).

The specific isolation of *E6-HPV16* oncogene from the nanoconstruct of commercial M-280 MPswith biotin-modified oligonucleotide complementary to oncogene prior to PCR amplification was realized. The detection of *E6-HPV16* gene using agarose gel electrophoresis showed a 2.6 fold higher fluorescence than the positive control (synthetic plasmid E6-HPV16-pUC57) (Figure 5). Thus, it was confirmed that using of commercial M-280 MPs increased the sensitivity of HPV-16 detection compared with the standard PCR method.

Moreover, three different “homemade” MPs that were prepared and characterized in our laboratory were used for isolation of the E6-HPV16-pUC57 plasmid to improve the PCR detection. These particles, MAN-37, MAN-127 and MAN-164, were characterized using SEM and Dynamic Light Scattering. The size of all tested MPs was increased due to the binding of DNA. The highest increase was observed while using MAN 37, which increased their size by 41%. MAN 127 increased their size by 32% and MAN-164 by 14%. This increase in the size of magnetic particles did not quantitatively correlate to the yield of DNA isolation. The yield was indeed highest for MAN-37, however no DNA was recovered using the MAN-127 (Figure 6G). The yield of DNA isolation was obtained using the fluorescence intensity detected on agarose gel 1.31 fold increase intensity in comparison with the control using magnetizable particles MAN-37 (Figure 6D). When the DNA isolation between these different MPs is compared, we can conclude that the yield using commercial M-280 MPs was twice higher than used MAN-37, which may be due to the imperfections in our “homemade” magnetizable particles. The yield DNA isolation was calculated using Adobe.photoshop (v. 8.0) software (Adobe, San Jose, CA, USA) for all these studies.

The second part of this work is based on the electrochemical detection of E6-HPV16 DNA after being released from the different magnetic nanoconstructs. There are many other studies about electrochemical biosensors for HPV DNA detection. Wide range of electrochemical techniques has been used for this aim such as DPV, SWV, AdTS SWV, CV, *etc.* [31,32,33,34,35,36,37,38,39].

The lowest concentration level of the analyte that is detectable above the noise of the system is called the detection limit (LOD) (considering 3 signal to noise ratio (*S*/*n* = 3) for all of assays in this work). The comparison between the detection limits of these different biosensors depends of the electrochemical techniques, type of the samples and type of electrode. With DPV method, Campos-Ferreira *et al.* (2013) [36] obtained a (LOD) of 18.13 nM using l-Cysteine gold electrode and Huang *et al.* (2011) [34] obtained 4.03 × 10^−5^ nM using a glassy carbon electrode modified with graphene gold nanorod polythionine. Wang *et al* (2012) [33] used SWCNT to detect hepatitis B and papillomavirus DNAs with a (LOD) of 3.47 × 10^−5^ nM. Bo *et al.* (2011) [38] using CV technique prepare DNA biosensors based on graphene obtained a (LOD)of 3.25 × 10^−4^ nM.

AdTS SWV was considered as the most sensitive electrochemical method for nucleic acid detection because the accumulation of biomolecules on the working electrode surface increases the sensitivity of the assay [21]. In our study we used SWV and AdTS SWV method. Two different E6-HPV16 DNA samples were obtained after their release from nanoconstructs using commercial M-280 MPs with a forward and reverse biotinylated oligonucleotide and these samples were analyzed by SWV method. The calibration curve of the *E6-HPV16* gene in concentration range was done from 0.15 to 2.5 µM. The detection limit (LOD) obtained for HPV DNA samples released from the construct was 0.2 nM and the limit of quantification (LOQ) was 0.7 nM. The higher DNA concentration was obtained using the forward oligonucleotide nanoconstruct (Figure 9). On the other hand, these differences in the measurements were not observed when using PCR after separation of nanoconstruct from commercial M-280 MPs using forward and reverse oligonucleotide (see Chapter 2.2 Isolation of E6-HPV16 Gene Using Commercial MPs with PCR Detection), because the detection by agarose gel electrophoresis with ethidium bromide staining was not a suitable method to show the differences between both oligonucleotides, while electrochemical measurements conferred higher specificity and sensitivity when compared with gel electrophoresis method.

The optimization of electrochemical detection of *E6-HPV16* gene and E6 primer was also characterized using AdTS SWV (Figure 7). The detection limit observed was of 1.43 × 10^−2^ nM for *E6-HPV16* gene and 4 × 27 × 10^−2^ nM for E6 oligonucleotide. Other studies applied also electrochemical HPV DNA detection, as Sabzi *et al.* (2008) [37] using SWV method coupled with methylene blue as an electroactive label on a pencil graphite electrode got a 0.5 nM of theLOD of DNA major capsid protein L1 gene. Jampasa *et al.* (2014) [32] obtained with the same method a LOD of 4.0 nM of DNA of type 16 with carbon electrodes. Zari *et al.* (2009) [39] also studied short DNA sequences of HPV treated with acid and characterized by SWV, they had a LOD 2 nM.

Our method showed more advantages because we obtained the highest sensitive DNA detection limit in our assays (0.2 nM for SWV and 1.43 × 10^−2^ nM for AdTS SWV) when compared with others SWV electrochemical methods. It clearly proved the advantages of working with MPs due to improve the HPV DNA isolation, showing a higher isolation and more sensitive technique than using just the standard PCR method.

## 4. Materials and Methods

### 4.1. Chemicals

All chemicals of ACS purity were obtained from Sigma-Aldrich (St. Louis, MO, USA) unless stated otherwise. The deionized water was prepared using reverse osmosis equipment Aqual 25 (Aqual s.r.o., Brno, Czech Republic). The deionized water was further purified by using apparatus MiliQ Direct QUV equipped with the UV lamp (MiliQ water, 18 MΩ, Millipore Corp., Billerica, MA, USA) was used in the course of the methodology for washing and rinsing processes. The pH was measured using pH meter WTW inoLab (Weilheim, Germany).

### 4.2. Cloning of the E6 Oncoprotein of Human Papillomavirus 16

The E6 gene of human papillomavirus 16 (GenBank accession number: BAN15931) was synthesized and cloned into the plasmid pUC57-Amp (GENEWIZ, South Plainfield, NJ, USA) resulting in pUC57 vector containing the *E6-HPV16* gene. The chemical transformation protocol was performed following the instructions of Invitrogen, using TOP10 chemically competent *E. coli* strain as host. Bacteria transformed with E6-HPV16-pUC57 plasmid were selected by ampicillin resistance. The positive transformants were confirmed by the PCR screening. The positive transformants were grown in LB (Luria-Bertani) broth with 50 µg·mL^−1^ ampicillin, shaking at 37 °C overnight.

### 4.3. Isolation and PCR Amplification of E6 Human Papillomavirus 16

The plasmid was purified using the Qiagen Miniprep Kit (Qiagen, Germantown, MD, USA) and the PCR amplification of the gene was performed using a set of primers flanking the complete open reading frame: 5′-ATGCACCAAAAGAGAACTGC-3′ (E6 forward) and 5′-TTACAGCTGGGTTTCTCTAC-3′ (E6 reverse). The PCR mixture (Taq PCR kit, New England Biolabs, Ispwich, MA, USA), contained the PCR buffer (10 mM Tris-HCl pH 8.3, 50 mM KCl with 1.5 mM MgCl_2_ included), 0.2 mM of dNTPs and 0.4 µM of each primers with E6-HPV16-pUC57 synthetic plasmid using as a template. The DNA amplification was carried out for 40 cycles of the denaturation at 94 °C for 30 s, the annealing at 56 °C for 30 s and the primer extension at 72 °C for 30 s. The amplified *E6-HPV16* oncogene was purified using MiniElute PCR Purification Kit (Qiagen, Germantown, MD, USA). The amplified product of 477 base pairs was analyzed by agarose gel electrophoresis and the conditions were as follows: 1% agarose gel (Agarose MP, Roche Diagnostics, Indianapolis, IN, USA) in TAE buffer, at 60 V for 160 min (Bio-Rad, Hercules, CA, USA). The 100 bp DNA ladder (New England Biolabs, Ispwich, MA, USA) was used as a molecule size marker. The ethidium bromide-labeled bands were visualized via UV transilluminator at 312 nm (Vilber-Lourmat, Marne-la-Valle’e Cedex, France).

### 4.4. Biotinylation of Oligonucleotides

Biotinylation was performed following the protocol of Biotin 3′ End DNA Labeling Kit (Thermo Scientific, Waltham, MA, USA). This procedure was optimized for labeling 5 pmol of 3′-OH ends in each reaction, using 1 µM oligonucleotide E6 forward (5′-GCAGTTCTCTTTTGGTGCAT-3′) and 1 µM oligonucleotide E6 reverse (5′-CATCTCTTTGGGTCGACATT-3′) complementary to the *E6-HPV16* gene sequence. Labeling efficiency was determined by dot blot technique (data not shown).

### 4.5. Magnetizable Particles and Their Preparation

10 mg·mL^−1^ of commercial Dynabeads M-280 Streptavidin (Invitrogen) in phosphate buffered saline (PBS) pH 7.4 with 0.09% sodium azide as a preservative were used in this study. The isolation protocol was performed according to the manufacturer’s instructions. The capture, washing and detection conditions were optimized. Our own magnetizable particles, MAN-37, MAN-127 and MAN-164, with the following characteristics, were also used in this experiment.

#### 4.5.1. MAN-37

NaBH_4_ (1 g) in 3.5% NH_3_ (50 mL) was added with stirring to a solution of Fe(NO_3_)_3_·9H_2_O (7.48 g) in water (400 mL). The mixture was heated at 100 °C for 2 h. The product was separated by external magnetic field and washed several times with water. Isopropyl alcohol (150 mL) was added, followed by 28% ammonia solution (20 mL) and tetraethyl orthosilicate (3.33 mL). This mixture was stirred and heated at 40 °C for 2 h. APTES (3.33 mL) was added and the mixture was heated for additional 1 h. The product was stirred overnight at 25 °C, separated by external magnetic field and washed several times with diluted ethanol (75%). Finally, the product was stored in 20 mL of ethanol (75%).

#### 4.5.2. MAN-127

Fe(NO_3_)_3_·9H_2_O (1.5 g) was dissolved in water (80 mL). Under stirring was added NaBH_4_ (0.2 g), dissolved in 10 mL of 3.5% NH_3_, and heated at 100 °C for 2 h. After cooling, the mixture was kept overnight at 25 °C and the maghemite was separated by external magnetic field, washed several times with water and water solution of polyvinylpyrrolidone (10 kDa) (0.2 g) was added with stirring. The mixture was stirred overnight, separated by external magnetic field, washed several times with water and left in 50 mL of PBS buffer pH 7.4.

#### 4.5.3. MAN-164

Fe(NO_3_)_3_·9H_2_O (1.5 g) was dissolved in water (80 mL). Under stirring was added NaBH_4_ (0.2 g), dissolved in 10 mL of 3.5% NH_3_, and heated at 100 °C for 2 h. After cooling, the mixture was kept overnight at 25 °C and magnetic nanoparticles were separated by external magnetic field, washed several times with water and water solution of sodium triphosphate (0.368 g) was added and the mixture was stirred for 2 h. Then, 1 M Ca(NO_3_)_2_·4H_2_O (6 mL) was poured into the solution, continued by stirring overnight. The modified maghemite was separated by external magnetic field, washed several times with water and stored in water (50 mL).

### 4.6. Characterization of Particle Size

The average particle size and size distribution were determined by quasielastic laser light scattering with a Malvern Zetasizer (NANO-ZS, Malvern Instruments Ltd., Worcestershire, UK). Then, 1.5 mL of solution of nanoparticles in distilled water (1 mg·mL^−1^) was put into a polystyrene latex cell and measured at a detector angle of 173°, a wavelength of 633 nm, a refractive index of 0.30, a real refractive index of 1.59, and a temperature of 25 °C.

### 4.7. Scanning Electron Microscopy (SEM)

The structures of our "homemade" MPs; MAN-37, MAN-127 and MAN-164 were also characterized by SEM using a MIRA3 LMU (Tescan, Brno, Czech Republic) equipped with a high brightness Schottky field emitter for low noise imaging at fast scanning rates. The SEM was fitted with TESCAN In-Beam SE detector. For automated acquisition of selected areas, a TESCAN proprietary software tool called Image Snapper was used. The software enabled automatic acquisition of selected areas with defined resolution. An accelerating voltage of 15 kV gave satisfactory results regarding maximum throughput.

### 4.8. Binding of DNA with Magnetizable Particles

#### 4.8.1. *E6-HPV16* Gene Isolation Using Commercial Dynabeads

One hundred microliters (10 mg·mL^−1^) of commercial Dynabeads M-280 Streptavidin was washed three times with Binding and washing (B & W) (2×) buffer containing 2 M NaCl; 1 mM EDTA; 10 mM Tris-HCl pH 7.5. Afterwards, to immobilize the nucleic acid 10 µL of oligonucleotide E6 forward (200 pmol) (5′-GCAGTTCTCTTTTGGTGCAT-3′), 10 µL of oligonucleotide E6 reverse (200 pmol) (5′-CATCTCTTTGGGTCGACATT-3′) and 100 µL of B & W (1×) buffer was added and incubated at 25 °C for 15 min on a rotator Multi RS-60 (Biosan Ltd., Riga, Latvia). Sample was washed 3× with 100 µL of B & W (1×) buffer on magnet. Subsequently, the *E6-HPV16* gene was isolated in a similar way. The 10 µL (100 µM) *E6-HPV16* gene was incubated at 25 °C for 30 min on a rotator and afterwards washed 3× with 100 µL of phosphate buffer (0.1 M NaCl; 0.05 M Na_2_HPO_4_; 0.05 M NaH_2_PO_4_) on magnet. The mixture was incubated at 25 °C for 30 min and washed on magnet 3× with phosphate buffer (3 × 100 µL). Afterwards, 10 µL of water in ACS purity was added and the mixture was incubated at 95 °C and 14000 rpm for 5 min on a Thermomixer Comfort (Eppendorf, Hamburg, Germany). Finally, samples were rapidly cooled on ice. MPs were separated by external magnetic field and the solution was used for subsequent measurements.

#### 4.8.2. Isolation of E6-HPV16-pUC57 Plasmid Using Laboratory-Synthesized Magnetized Particles

Three different "homemade" MPs (MAN-37, MAN-127 and MAN-164) were used for the isolation. Protocol of isolation was as follows: 8 µL of particles in appropriate storage solution was mixed with 10 µL of plasmid (10 µg·mL^−1^) and 70 µL of binding buffer containing 10 µL of 750 mM sodium acetate pH 6 and 60 µL of 85% ethanol. The samples were vigorously vortexed. After application of external magnetic field for 3 min, the debris was removed and MPs with bound plasmid were washed using 150 µL of 85% ethanol. The MPs with bound plasmid were resuspended in binding buffer and water to the original volume and in some of the experiments aliquots were taken for later on PCR on particles. The MPs with bound plasmid were again washed using 150 µL of 85% ethanol and 25 µL of 10 mM Tris-HCl pH 8 was added to elute the plasmid molecules from particle surface. The samples were vigorously vortexed and an external magnetic field was applied for 3 min. Eluate was removed and used for PCR amplification of *E6-HPV16* gene in isolated plasmid. For PCR, 5 µL of eluate and 5 or 0.5 µL of MPs-plasmid was used. As a positive control, 0.5 E6-HPV16-pUC57 µL of plasmid was used.

### 4.9. Electrochemical Analysis

The instrumentations for electrochemical measurement were used as follows: AUTOLAB PGS30 Analyzer (EcoChemie, Amsterdam, The Netherlands) connected to a 663 VA Stand (Metrohm, Herissau, Switzerland). Standard electrochemical cell was equipped with three electrodes. A hanging mercury drop electrode with a drop area of 0.4 mm^2^ was employed as the working electrode. An Ag/AgCl/3 M KCl electrode as the reference and platinum electrode as auxiliary was used. All samples were measured by AdTS SWV method. An acetate buffer pH 5.0 (0.2 M CH_3_COONa, CH_3_COOH) was used as a supporting electrolyte. For smoothing and baseline correction, the software GPES 4.9 supplied by EcoChemie was employed.

#### 4.9.1. Optimization of Electrochemical Detection of CA Peak in Product of Amplification

Using AdTS SWV method CA peak was determined at potential (−1.40 ± 0.03 V). SWV method was enriched with AdTS technique for the purposes of accumulation of biologically active substances on the surface of the dropping mercury working electrode. Prior to measurements, all samples were deoxygenated by argon (99.999%) for 120 s. Parameters of AdTS SWV were follows: initial potential of 0.0 V, end potential of −1.8 V, potential step of 0.005 V, amplitude of 0.025 V, frequency of 280 Hz. Two other parameters (frequency and time of accumulation) were optimized.

#### 4.9.2. Detection of CA Peak in the Product of PCR Amplification of *E6-HPV16* Gene

PCR products obtained after 10, 20, 30, 35, and 40 cycles of PCR of E6-HPV16-pUC57 plasmid were studied by electrochemistry. Data for calibration curve, reflecting the dependence of the relative peak height (%) on the number of PCR cycles, were obtained.

## 5. Conclusions

In this work the *E6-HPV16* gene was isolated and electrochemically characterized, after being released from commercial (M-280 Streptavidin) and "homemade" (MAN-37, MAN-127 and MAN-164) magnetizable particles. A highest yield of DNA isolation was observed when using combination of PCR standard method with particles M-280 and MAN-37. Based on our results, it can be concluded that magnetizable particles can serve as an effective tool in therapy and diagnostic of the carcinogenesis. Due to their perfect attributes, non-toxicity and possibility of easy modification, and applications into automated assay, they could be utilized for large scales of clinical methods. In our future work, we will develop a microfluidic chip based on the extraction of magnetizable particles in the presence of a special fixed magnet with a permanent magnetic field, coupled to electrochemical assays that resulting in an improvement of nucleic acid detection of viruses or other pathogens agents.

## Abbreviations

AdTS	Adsorptive transfer stripping
APTES	3-aminopropyltriethoxysilane
CA	Cytosine-adenine
CV	Cyclic voltammetry
DPV	Differential pulse voltammetry
EV	Elimination voltammetry
HPV	Human papillomavirus
MPs	Magnetizable particles
PBS	phosphate buffered saline
PCR	Polymerase chain reaction
SEM	Scanning electron microscopy
SWV	Square wave voltammetry
